# The prehospital quick SOFA score is associated with in-hospital mortality in noninfected patients: A retrospective, cross-sectional study

**DOI:** 10.1371/journal.pone.0202111

**Published:** 2018-08-16

**Authors:** Osamu Kitahara, Kei Nishiyama, Bunsei Yamamoto, Shigeaki Inoue, Sadaki Inokuchi

**Affiliations:** 1 Department of Emergency Medicine, Tokai University Hachioji Hospital, Hachioji, Tokyo, Japan; 2 Critical Care Center, National Hospital Organization Kyoto Medical Center, Kyoto, Japan; 3 Department of Emergency and Critical Care Medicine, Tokai University School of Medicine, Isehara, Kanagawa, Japan; Azienda Ospedaliero Universitaria Careggi, ITALY

## Abstract

This study aimed to determine the accuracy of the quick Sequential Organ Failure Assessment (qSOFA) score in predicting mortality among prehospital patients with and without infection. This single-center, retrospective, cross-sectional study was conducted among patients who arrived via the emergency medical services (EMS). We calculated the qSOFA score and Modified Early Warning Score (MEWS) from prehospital records. We identified patients as infected if they received intravenous antibiotics at the emergency department or within the first 24 hours. Receiver operating characteristic analysis was used to evaluate and compare the performance of the qSOFA score, each physiological parameter, and the MEWS in predicting admission and in-hospital mortality in patients with and without infection. Multivariate analysis was used to evaluate the qSOFA score and other risk factors. Out of 1574 prehospital patients, 47.1% were admitted and 3.2% died in the hospital. The performance of the qSOFA score in predicting in-hospital mortality in noninfected patients was 0.70, higher than for each parameter and the MEWS. The areas under the curve for the qSOFA+ model vs. the qSOFA- model was 0.77 vs. 0.68 for noninfected patients (p <0.05) and 0.71 vs. 0.68 for infected patients (p = 0.41). The likelihood ratio test comparing the qSOFA- and qSOFA+ groups demonstrated significant improvement for noninfected patients (p <0.01). Multivariate regression analysis for in-hospital mortality demonstrated that the qSOFA score is an independent prognosticator for in-hospital mortality, especially among noninfected patients (odds ratio, 3.60; p <0.01). In conclusion, the prehospital qSOFA score was associated with in-hospital mortality in noninfected patients and may be a beneficial tool for identifying deteriorating patients in the prehospital setting.

## Introduction

Physiological parameters are indicators of the patient’s health condition and are routinely used by emergency medical service (EMS) providers. In the prehospital setting, early identification of high-risk patients is essential to transfer them appropriately and possibly to allow early intervention of emergency department (ED) staff. Scoring systems for vital signs such as the Modified Early Warning Score (MEWS) and National Early Warning Score (NEWS) have been developed for the hospital setting and are currently used in the prehospital setting to identify patients who require intensive care unit (ICU) admission, have adverse in-hospital events, or mortality [1–3].

The quick Sequential Organ Failure Assessment (qSOFA) score is a new tool for identifying critically-ill infected patients outside the ICU [4]. The qSOFA score is based on only three parameters: respiratory rate, systolic blood pressure, and any alteration in mental status. Recently, the qSOFA score was reported to be useful in predicting mortality in ED patients with and without infection [5]. However, few studies have evaluated the utility of the qSOFA score in the prehospital setting.

This study aimed to investigate the accuracy of the qSOFA score in predicting the risk of admission and mortality in prehospital patients with and without infection.

## Materials and methods

The study protocol was approved by the institutional review board of Tokai University School of Medicine (#17R-107) and patient consent was exempted because of the retrospective nature. All data were fully anonymized before we analyzed them in this retrospective study.

### Study design and participants

This is a single-center, retrospective, cross-sectional study of patients presenting to the ED of Tokai University Hachioji Hospital (a 500-bed, general hospital obtained the stroke and ST-elevated myocardial infarction receiving center) in Tokyo, Japan. The ED serves a population of 0.55 million. All patients who arrived at the ED in ambulances from January 1 to June 30, 2016, were enrolled. We gathered data from prehospital records that documented initial vital signs and hospital electronic medical records. Patients with young age (<18 years), cardiopulmonary arrest, and missing information were excluded. We identified the patients as infected if they received antibiotics for suspicion of infection at the ED or within the first 24 hours.

### Measurements

We calculated the qSOFA score and MEWS from prehospital records of blood pressure, heart rate, body temperature, oxygen saturation, and respiratory rate. The components of qSOFA were blood pressure ≤100 mmHg, respiratory rate ≥22 breaths/min, and altered mental status (S1 Table). A Japan Coma Scale score (range: 0–300 points) of <1 (equivalent to a Glasgow Coma Scale score of <15 points) was used in estimating the qSOFA score (Table 1) [6]. The MEWS is based on five basic physiological parameters (the AVPU [alert, voice, pain, unresponsive] score, respiratory rate, heart rate, systolic blood pressure, and body temperature) (S2 Table) [7].

**Table 1:** Japan Coma Scale.

Conscious Level	Featuers	Scale
awake without any stimuli	alert	0
	almost fully counscious but not normal	1
	unable to recognize time, place, person	2
	unable to recall name or date of birth	3
arousable but reverts to previous state if stimulus stops	easily by being spoken to	10
	with loud voice	20
	by painful stimuli	30
unarousable	responds to avoid the stimuli	100
	responds with slight movements	200
	dose not respond at all	300

### Outcomes

The primary outcome was in-hospital mortality, which was defined as death during the hospital stay as documented in the medical record. The secondary outcome was hospital admission.

### Statistical analysis

Baseline characteristics were presented as medians and interquartile ranges for continuous variables and as number of patients for categorical variables. The Kruskal-Wallis test was performed to compare four score groups (qSOFA: 0, 1, 2, and 3) for continuous variables, and the chi-square test was used for categorical variables. To predict in-hospital mortality, each qSOFA score point and the MEWS were assessed using sensitivity, specificity, predictive values, and likelihood ratios. Receiver operating characteristic (ROC) analysis was employed to evaluate and compare the performance of the qSOFA score, each physiological parameter, and the MEWS in predicting the admission and in-hospital mortality of patients with and without infection. Logistic regression models were used to evaluate risk factors (age, sex, qSOFA score) with multivariate analyses. We used two multivariate analysis models and performed the likelihood ratio test and ROC analysis to evaluate these models in the prediction of mortality. The model that included age, sex, and qSOFA score (qSOFA+) as independent variables was compared with the other model that consisted of age and sex without qSOFA score (qSOFA-). A two-tailed p value of <0.05 was considered statistically significant. All statistical analyses were performed using R version 3.4.1 (The R Development Core Team, Vienna, Austria).

## Results

### Characteristics of study subjects

**Fig 1:**
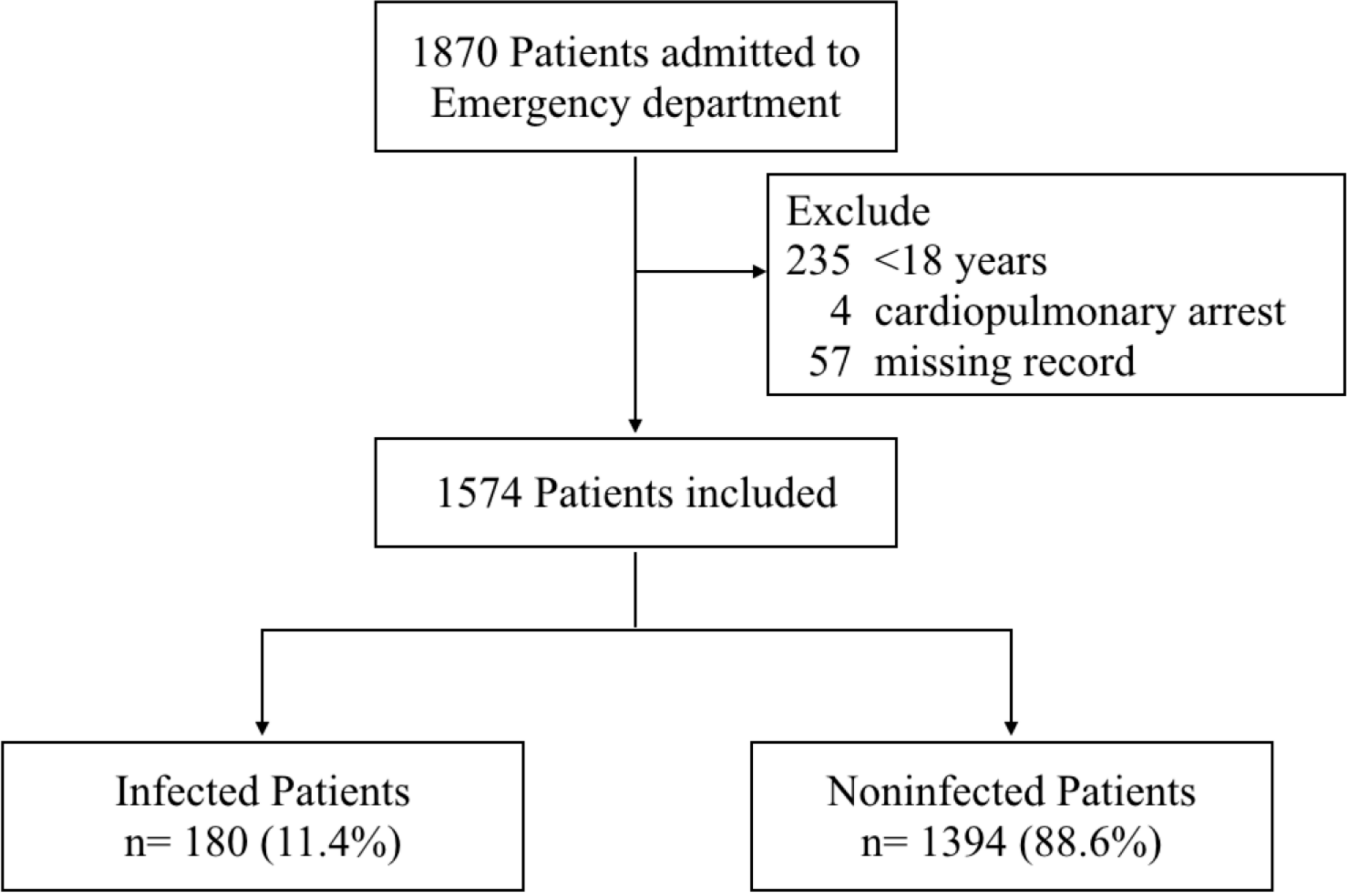
Flowchart of patients enrolled in the study.

A total of 1870 patients presented to the ED via the EMS during the study period. Patients who were younger than 18 years (n = 235), had cardiopulmonary arrest (n = 4), and/or had missing values required for calculating the qSOFA score and MEWS (n = 57) were excluded. Thus, 1574 patients were included in the analysis (Fig 1). Among them, 88.6% were patients who were not infected. The median age was 72 years (interquartile range, 55–81 years), and 54.3% were male. For all patients, infected patients, and noninfected patients, the admission rates were 47.1% (n = 741), 92.8% (n = 167) and 41.2% (n = 574), and the in-hospital mortality rates were 3.2% (n = 51), 11.1% (n = 20) and 2.2% (n = 31), respectively. Among all the patients, 821 (52.2%), 625 (39.7%), 113 (7.2%), and 15 (1%) had qSOFA scores of 0, 1, 2, and 3, respectively. The baseline characteristics and primary outcome are summarized in Table 2.

**Table 2:** Patient’s characteristics and outcomes.

	All patients (n = 1574)	Infected patients (n = 180)	Noninfected patients (n = 1394)	p
Age median (IQR), y	72 (55–81)	79 (67–85)	71 (53–80)	<0.001
Male, No, (%)	855 (54.3)	103 (57.2)	752 (53.9)	0.453
JCS, median (IQR)	0 (0–1)	0 (0–3)	0 (0–1)	<0.001
AVPU, n (%)				0.06
A	1496 (95.0)	165 (91.7)	1331 (95.5)	
V	54 (3.4)	9 (5.0)	45 (3.2)	
P	22 (1.4)	5 (2.8)	17 (1.2)	
U	2 (0.1)	1 (0.6)	1 (0.1)	
Respiratory rate, median (IQR), breaths/min	18 (18–24)	18 (18–24)	18 (18–24)	<0.001
Heart rate median, median (IQR), beats/min	84 (72–102)	102 (89–114)	84 (72–102)	<0.001
Systolic blood pressure, median (IQR), mmHg	137 (118–160)	124 (110–147)	138 (120–160)	<0.001
Temperature, median (IQR), °C	36.6 (36.2–36.9)	37.6 (36.8–38.6)	36.5 (36.2–36.8)	<0.001
qSOFA, n (%)				<0.001
0	821 (52.2)	66 (36.7)	755 (54.2)	
1	625 (39.7)	71 (39.4)	554 (39.7)	
2	113 (7.2)	37 (20.6)	76 (5.5)	
3	15 (1.0)	6 (3.3)	9 (0.6)	
MEWS, n (%)				<0.001
0	1 (0.1)	0 (0.0)	1 (0.1)	
1	679 (43.1)	34 (18.9)	645 (46.3)	
2	401 (25.5)	37 (20.6)	364 (26.1)	
3	247 (15.7)	33 (18.3)	214 (15.4)	
4	126 (8.0)	33 (8.3)	93 (6.7)	
5	66 (4.2)	21 (11.7)	45 (3.2)	
6	30 (1.9)	9 (5.0)	21 (1.5)	
7	18 (1.1)	9 (5.0)	9 (0.6)	
8	6 (0.4)	4 (2.2)	2 (0.1)	
Infection, n (%)	180 (11.4)	180 (100.0)	0 (0.0)	<0.001
Trauma, n (%)	209 (13.3)	0 (0.0)	209 (15.0)	<0.001
Stroke, n (%)	97 (6.2)	0 (0.0)	97 (7.0)	<0.001
Peripheral vertigo or suspected, n (%)	94 (6.0)	0 (0.0)	94 (6.7)	<0.001
Acute alcohol intoxication, n (%)	88 (6.0)	0 (0.0)	88 (6.3)	<0.001
Acute coronary syndrome, n (%)	60 (3.8)	0 (0.0)	60 (4.3)	0.009
Others n (%)	846 (53.7)	0 (0.0)	846 (60.7)	<0.001
Admission, n (%)	741 (47.1)	167 (92.8)	574 (41.2)	<0.001
Inhospital death, n (%)	51 (3.2)	20 (11.1)	31 (2.2)	<0.001

IQR, interquartile range; JCS, Japan Coma Scale; MEWS, Modified Early Warning Score; qSOFA, quick Sequential Organ Failure Assessment

### Main results

Table 3 summarizes the sensitivity, specificity, predictive values, and likelihood ratios of predicting mortality across the qSOFA scores and MEWS. The positive likelihood ratio was higher for a qSOFA score of 3 (26.13; 95% confidence interval [CI], 9.85–69.29) than for other qSOFA scores and MEWS.

**Table 3:** Summary statistics of accuracy for predicting mortality.

	Sensitivity (95% CI)	Specificity(95% CI)	PPV (95% CI)	NPV (95% CI)	LR+ (95% CI)	LR- (95% CI)
qSOFA						
1≦	78.4 (64.7–88.7)	53.2 (50.6–55.7)	5.3 (3.8–7.2)	98.7 (97.6–99.3)	1.68 (1.44–1.95)	0.41 (0.24–0.69)
2≦	31.4 (19.1–45.9)	92.6 (91.2–94)	12.5 (7.3–19.5)	97.6 (96.6–98.3)	4.27 (2.74–6.65)	0.74 (0.62–0.89)
3≦	13.7 (5.7–26.3)	99.5 (99–99.8)	46.7 (21.3–73.4)	97.2 (96.2–97.9)	26.13 (9.85–69.29)	0.87 (0.78–0.97)
MEWS						
1≦	100 (89.7–100)	1 (0–0.4)	3.2 (2.4–4.2)	100 (1.3–100)	1 (0.03–0.04)	0
2≦	76.5 (62.5–87.2)	43.9 (41.3–46.4)	4.4 (3.1–5.9)	98.2 (96.9–99.1)	1.36 (1.16–1.6)	0.54 (0.33–0.88)
3≦	51.0 (36.6–65.2)	69.3 (67–71.6)	5.3 (3.5–7.6)	97.7 (96.6–98.5)	1.66 (1.26–2.2)	0.71 (0.53–0.94)
4≦	35.3 (22.4–49.9)	85 (83.1–86.8)	7.3 (4.4–11.3)	97.5 (96.5–98.3)	2.36 (1.6–3.48)	0.76 (0.62–0.93)
5≦	25.5 (14.3–39.6)	93 (91.6–94.2)	10.8 (5.9–17.8)	97.4 (96.4–98.1)	3.63 (2.19–6)	0.8 (0.68–0.94)
6≦	15.7 (7–28.6)	97 (96–97.8)	14.8 (6.6–27.1)	97.2 (96.2–97.9)	5.14 (2.59–10.43)	0.87 (0.77–0.98)
7≦	5.9 (1.2–16.2)	98.6 (97.9–99.1)	12.5 (2.7–32.4)	96.9 (95.9–97.7)	4.27 (1.31–13.8)	0.95 (0.89–1.02)
8≦	2 (0–10.4)	99.7 (99.2–99.9)	16.7 (0.4–64.1)	96.8 (95.8–97.6)	5.97 (0.71–50.2)	0.98 (0.95–1.02)

LR, Likelihood ratio; MEWS, Modified Early Warning Score; NPV, Negative predictive value; PPV, Positive predictive value; qSOFA,quick Sequential Organ Failure Assessment

**Fig 2:**
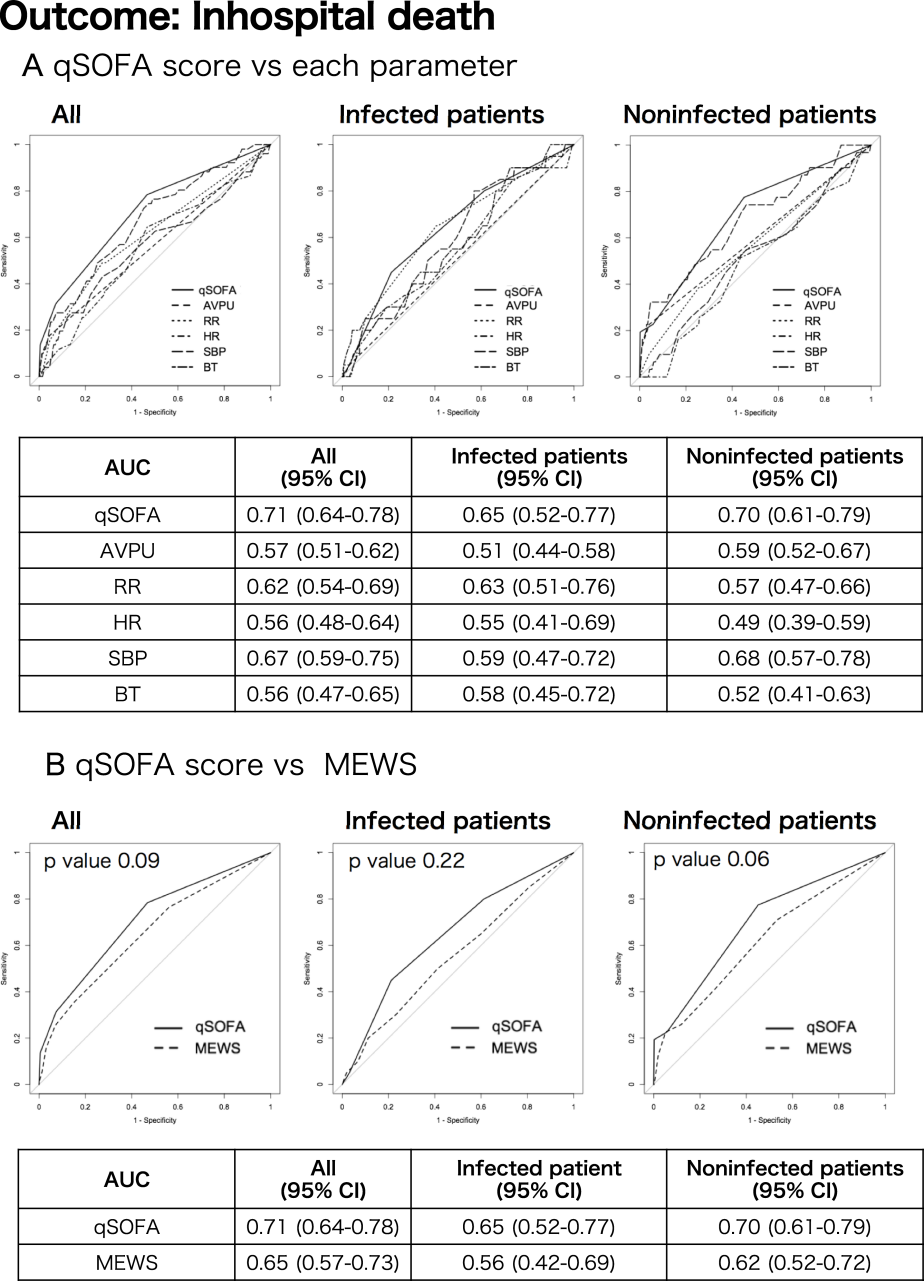
Receiver operating characteristic curves for mortality. (A) qSOFA score vs. each parameter in all patients, noninfected patients, and infected patients; (B) qSOFA score vs. MEWS in the three groups. AUC, the area under the curve; BT, body temperature; HR, heart rate; MEWS, Modified Early Warning Score; qSOFA, quick Sequential Organ Failure Assessment; RR, respiratory rate; SBP, systolic blood pressure.

The results of the ROC analysis for admission in all patients, infected patients, and noninfected patients are shown in S1 Fig. The performance of each score and parameter in all three groups was low (Range of the areas under the curve [AUCs], 0.47–0.60). ROC curves for predicting in-hospital mortality were also evaluated (Fig 2). In all patients, AUCs for qSOFA score vs. MEWS were 0.71 (95% CI, 0.64–0.78) vs. 0.65 (95% CI, 0.57–0.73) (p = 0.09). The respective AUCs were 0.65 (95% CI, 0.52–0.77) vs. 0.56 (95% CI, 0.42–0.69) in infected patients (p = 0.22), and 0.70 (95% CI, 0.61–0.79) vs. 0.62 (95% CI, 0.52–0.72) in noninfected patients (p = 0.06). The AUCs for qSOFA score in the three groups, especially in all patients and noninfected patients, were higher than for each parameter and the MEWS.

**Fig 3:**
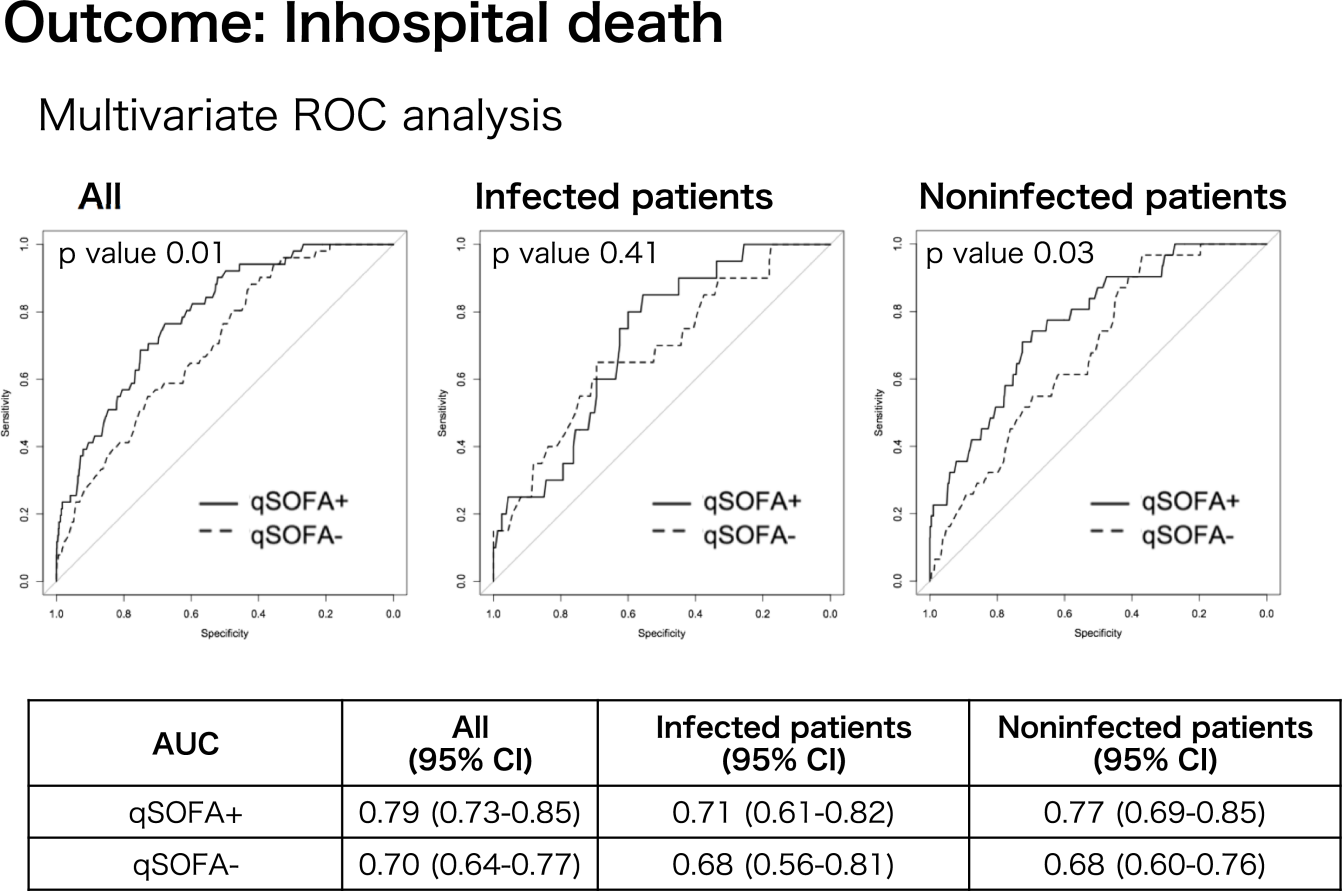
Multivariate receiver operating characteristic curve analysis for mortality in all patients, noninfected patients, and infected patients. The qSOFA+ model was based on the risk factors of age, sex, and qSOFA score. The qSOFA- model was based on age and sex only. AUC, the area under the curve; qSOFA, quick Sequential Organ Failure Assessment.

The AUCs for the qSOFA+ model vs. the qSOFA- model were 0.79 (95% CI, 0.73–0.85) vs. 0.70 (95% CI, 0.64–0.77) in all patients, 0.77 (95% CI, 0.69–0.85) vs. 0.68 (95% CI, 0.60–0.76) in noninfected patients, and 0.71 (95% CI, 0.61–0.82) vs. 0.68 (95% CI, 0.56–0.81) in infected patients (Fig 3). In all patients and noninfected patients, both models were statistically significant (p <0.01 and p <0.05, respectively), suggesting that the qSOFA score increases the AUC, especially in noninfected patients. In the likelihood ratio test comparing qSOFA- and qSOFA+ in the three groups, while the qSOFA+ model did not show significant improvement in infected patients, it indicated a significant improvement in all and noninfected patients (both p <0.01) (Table 4).

**Table 4:** Likelihood ratio test comparing models for predicting mortality.

qSOFA- vs qSOFA+	L.R.χ^2^	d.f.	P-value
All	37.9	1.0	<0.001
Infected patinets	3.7	1.0	0.052
Noninfected patients	27.6	1.0	<0.001

d.f., degrees of freedom; L.R., likelihood ratio; qSOFA, quick Sequential Organ Failure Assessment

Table 5 shows the multivariate regression analysis for mortality in the three groups. The odds ratios of qSOFA score were 3.13 (95% CI, 2.19–4.48, p <0.01) in all patients, 3.60 (95% CI, 2.26–5.72, p <0.01) in noninfected patients, and 1.76 (95% CI, 0.99–3.13, p <0.01) in infected patients. These results showed that qSOFA score is an independent prognostic factor for in-hospital mortality, especially in noninfected patients.

**Table 5:** Multivariate regression analysis for mortality.

**All patients**			
	Odds Ratio	95% CI for OR	p Value
Age	1.04	1.02–1.07	<0.01
Male	2.68	1.42–5.08	<0.01
qSOFA	3.13	2.19–4.48	<0.01
**Noninfected patients**			
	Odds Ratio	95% CI for OR	p Value
Age	1.04	1.01–1.07	<0.01
Male	2.32	1.05–5.10	<0.01
qSOFA	3.60	2.26–5.72	<0.01
**Infected patients**			
	Odds Ratio	95% CI for OR	p Value
Age	1.04	1.00–1.08	0.06
Male	3.00	1.00–8.99	0.05
qSOFA	1.76	0.99–3.13	0.05

qSOFA, quick Sequential Organ Failure Assessment

## Discussion

In this study, we found that the prehospital qSOFA score was associated with in-hospital mortality in noninfected patients compared to the MEWS and physiological parameters.

Although the qSOFA score is a tool for identifying infected patients with high-risk outcomes outside the ICU, few studies have investigated its utility in the prehospital setting. In recent studies, the performance of the qSOFA score in predicting complications was evaluated only in prehospital patients with infection [8–10]. To our knowledge, this study is the first to investigate the prediction of mortality in prehospital patients regardless of the presence of infection.

Here, the performance of the qSOFA score in association with in-hospital mortality was useful in noninfected patients compared with infected patients. In a retrospective study conducted in the ED setting, Singer et al. found that qSOFA scores were associated with in-hospital mortality in patients with and without infection [5]. However, the average age and mortality of the patients in their study were lower than those in our study. Furthermore, they excluded fast-track care, dental, psychiatric, and labor and delivery patients. Similar to our study, it was reported that the predictive ability of the qSOFA score in predicting complications in prehospital patients with infection was not satisfactory [10]. Moreover, in the ED setting, the performance of the qSOFA score had low accuracy in predicting mortality among critically ill septic patients [11]. Considering such findings, the qSOFA score is therefore not sufficient for predicting mortality in infected patients in the prehospital setting.

Early warning scores (EWS) such as the MEWS and NEWS are also tools for identifying patients at risk for critical illness outside the ICU. The MEWS is based on five physiological parameters and used in the United States and Europe [7]. Meanwhile, the NEWS is based on seven components (six vital signs and supplemental oxygen) and used in the United Kingdom [12]. Several studies have evaluated the prediction of mortality in prehospital patients. In a retrospective study by Fullerton et al., the authors showed that the MEWS in the prehospital setting was associated with adverse outcomes [1]. The AUC was 0.799 (95% CI, 0.738–0.856). Silcock et al. also showed that the NEWS in the prehospital setting was associated with mortality [2]. They established that the AUC for 30-day mortality was 0.740 (95% CI, 0.661–0.819). These results suggest that EWS are useful in predicting mortality. However, it may be difficult and complex for EMS staff to calculate EWS in a busy and stressful environment. A previous study showed that errors occurred by calculating EWS manually [13]. In contrast, the qSOFA score is a simple tool that can be quickly calculated without tables and laboratory tests because it comprises only three parameters (systolic blood pressure, alteration in mental status, and respiratory rate). We also conducted an additional multivariate analysis to evaluate risk factors by the addition of other parameters (heart rate and oxygen saturation). The results were consistent with our finding that qSOFA score is an independent prognosticator of in-hospital mortality in all patients and noninfected patients (data not shown).

This study has some limitations. First, it was a single-center, retrospective study, so the results cannot be generalized. Also, our hospital is not a tertiary care hospital; thus, it was not possible to transfer severe sepsis or septic shock patients to our hospital. The mortality rate in a tertiary care hospital in Japan is very high (9.1%) comprising 56% trauma patients; however, it includes only 3% infected patients [14]. Our study population is similar to that of Singer et al., which comprised 18% infected patients with 1.6% in-hospital mortality. Large multicenter, prospective studies are thus required to confirm our results. Second, the AVPU score and MEWS were calculated retrospectively from prehospital records by three ED physicians. In the original study, the qSOFA score was evaluated according to the Glasgow Coma Scale [4]. However, in Japan, EMS staff use the Japan Coma Scale score to evaluate the patient’s level of consciousness. Lastly, we defined patients as infected based on the administration of intravenous antibiotics only, while one study included patients who received both oral and intravenous antibiotics as suspected infected patients [4]. Thus, we might have underestimated the number of infected patients. To refine the identification of infected patients, we distinguished between proven infection (culture positive and/or clinically obvious infection) and suspected infection among 180 infected patients. The number of proven infected patients who were culture “positive” and/or “clinically obvious infected cases” was 120 (66.7%) (data not shown). We also conducted subgroup analysis comprising both patients with proven infection and patients with suspected infection; however, the results were consistent with our main findings (data not shown).

## Conclusion

We found that this tool was not sufficient for predicting mortality in infected patients. Nevertheless, the prehospital qSOFA score was more accurate than the MEWS and physiological parameters in predicting in-hospital mortality in noninfected patients. Further multicenter, prospective studies may be required to achieve more accurate results.

## Supporting information

S1 Table Quick Sequential Organ Failure Assessment (qSOFA) score. (PDF)

S2 Table Modified Early Warning Score (MEWS). (PDF)

S1 Fig Receiver operating characteristic curves for admission. (A) qSOFA score vs. each physiologic parameter in all patients, noninfected patients, and infected patients; (B) qSOFA score vs. MEWS in the three groups. AUC, the area under the curve; BT, body temperature; HR, heart rate; MEWS, Modified Early Warning Score.
(PDF)
