# Supplementary material for: The prehospital quick SOFA score is associated with in-hospital mortality in noninfected patients: A retrospective, cross-sectional study
Source: PLoS One. 2018 Aug 16;13(8):e0202111. doi: 10.1371/journal.pone.0202111 (PMC6095537; doi:10.1371/journal.pone.0202111)
Supplement: S1 Table — (PDF) [file pone.0202111.s001.pdf]

S1 Table . quick Sequential Organ Failure Assessment (qSOFA) score

parameter	point
respiratory rate ≥22 breaths/min	1
systolic blood pressure ≤100mmHg	1
altered metal status GCS<15	1
