# Supplementary material for: The prehospital quick SOFA score is associated with in-hospital mortality in noninfected patients: A retrospective, cross-sectional study
Source: PLoS One. 2018 Aug 16;13(8):e0202111. doi: 10.1371/journal.pone.0202111 (PMC6095537; doi:10.1371/journal.pone.0202111)
Supplement: S2 Table — (PDF) [file pone.0202111.s002.pdf]

S2 Table . Modified Early Warning Score (MEWS)

	3	2	1	0	1	2	3
Respiratory rate (breaths/min)		<9		9-14	15-20	21-29	≥30
Heart rate (beats/min)		<40	41-50	51-100	101-110	111-129	≥130
Systolic blood pressure (mmHg)	<70	71-80	81-100	101-199		≥200	
Temperature (℃)		<35		35-38.4		≥38.5	
Coscious level (AVPU score)				Alert	Reacting to Voice	Reacting to Pain	Unresposible
