# Supplementary material for: The prehospital quick SOFA score is associated with in-hospital mortality in noninfected patients: A retrospective, cross-sectional study
Source: PLoS One. 2018 Aug 16;13(8):e0202111. doi: 10.1371/journal.pone.0202111 (PMC6095537; doi:10.1371/journal.pone.0202111)
Supplement: S1 Fig — (A) qSOFA score vs. each physiologic parameter in all patients, noninfected patients, and infected patients; (B) qSOFA score vs. MEWS in the three groups. AUC, the area under the curve; BT, body temperature; HR, heart rate; MEWS, Modified Early Warning Score. (PDF) [file pone.0202111.s003.pdf]

Outcome: admission

A qSOFA score vs each parameter

All | Infected patients | Noninfected patients

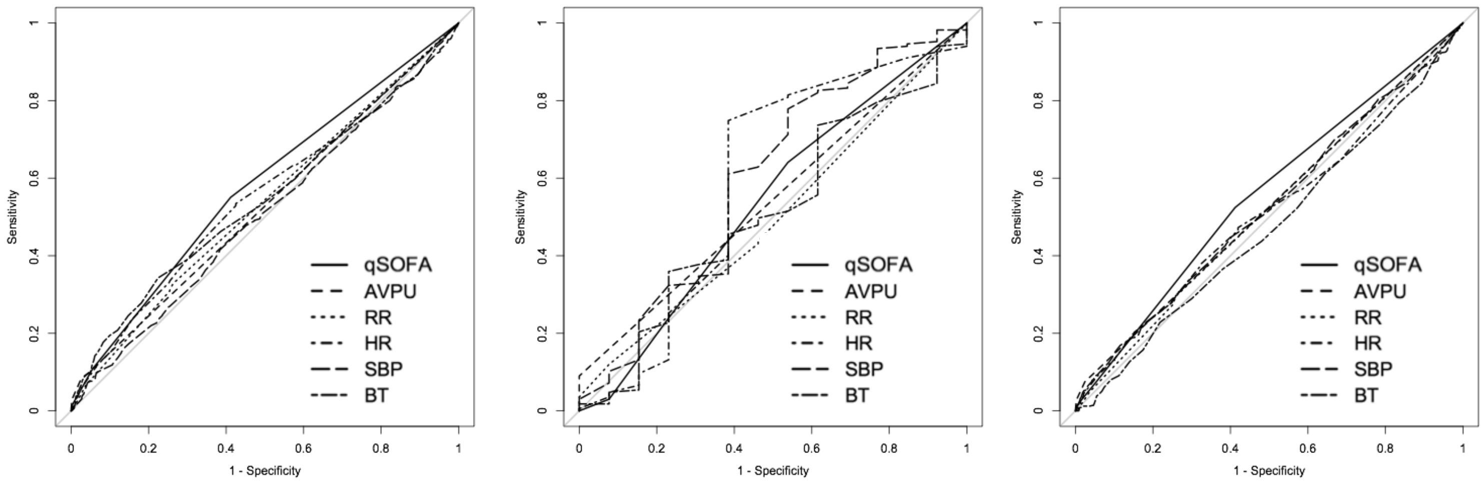

AUC	All (95% CI)	Infected patients (95% CI)	Noninfected patients (95% CI)
qSOFA	0.58 (0.55-0.60)	0.54 (0.37-0.71)	0.56 (0.53-0.59)
AVPU	0.53 (0.52-0.54)	0.55 (0.52-0.57)	0.53 (0.51-0.54)
RR	0.53 (0.51-0.56)	0.50 (0.36-0.64)	0.52 (0.49-0.54)
HR	0.55 (0.52-0.58)	0.59 (0.39-0.79)	0.51 (0.48-0.54)
SBP	0.50 (0.48-0.53)	0.60 (0.41-0.78)	0.52 (0.49-0.55)
BT	0.54 (0.51-0.57)	0.51 (0.34-0.68)	0.47 (0.44-0.51)

B qSOFA score vs MEWS

All | Infected patients | Noninfected patients

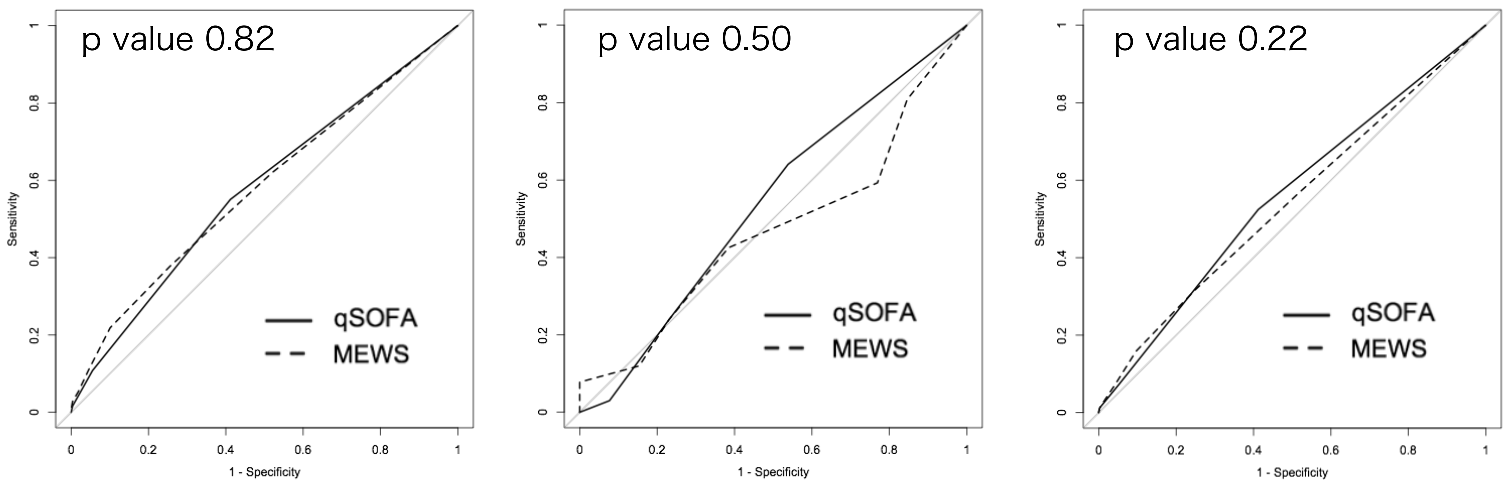

AUC	All (95% CI)	Infected patients (95% CI)	Noninfected patients (95% CI)
qSOFA	0.58 (0.55-0.60)	0.54 (0.37-0.71)	0.56 (0.53-0.59)
MEWS	0.58 (0.55-0.60)	0.47 (0.32-0.62)	0.54 (0.51-0.57)
